# Cloning, Characterization and Effect of *TmPGRP-LE* Gene Silencing on Survival of *Tenebrio Molitor* against *Listeria monocytogenes* Infection

**DOI:** 10.3390/ijms141122462

**Published:** 2013-11-14

**Authors:** Hamisi Tindwa, Bharat Bhusan Patnaik, Dong Hyun Kim, Seulgi Mun, Yong Hun Jo, Bok Luel Lee, Yong Seok Lee, Nam Jung Kim, Yeon Soo Han

**Affiliations:** 1 Division of Plant Biotechnology, Institute of Environmentally-Friendly Agriculture (IEFA), College of Agriculture and Life Sciences, Chonnam National University, Gwangju 500-757, Korea; E-Mails: tindwa@yahoo.com (H.T.); drbharatbhusan4@gmail.com (B.B.P.); kjsdh3@hanmail.net (D.H.K.); tori1127@nate.com (S.M.); yhun1228@chonnam.ac.kr (Y.H.J.); 2 National Research Laboratory of Defense Proteins, College of Pharmacy, Pusan National University, Jangjeon Dong, Kumjeong Ku, Busan 609-735, Korea; E-Mail: brlee@pusan.ac.kr; 3 Department of Life Science and Biotechnology, College of Natural Sciences, Soonchunhyang University, Asan City 336-745, Korea; E-Mail: yslee@sch.ac.kr; 4 Division of Applied Entomology, National Academy of Agricultural Science, Rural Development Administration, 61th, Seodun-dong, Gwonseon-gu, Suwon, Gyeonggi-do 441-853, Korea; E-Mail: vastnj@korea.kr

**Keywords:** peptidoglycan recognition protein, *Tenebrio molitor*, *Listeria monocytogenes*, PGRP domain, RNA interference

## Abstract

Peptidoglycan recognition proteins (PGRPs) are a family of innate immune molecules that recognize bacterial peptidoglycan. PGRP-LE, a member of the PGRP family, selectively binds to diaminopimelic acid (DAP)-type peptidoglycan to activate both the immune deficiency (Imd) and proPhenoloxidase (proPO) pathways in insects. A PGRP-LE-dependent induction of autophagy to control *Listeria monocytogenes* has also been reported. We identified and partially characterized a novel PGRP-LE homologue, from *Tenebrio molitor* and analyzed its functional role in the survival of the insect against infection by a DAP-type PGN containing intracellular pathogen, *L. monocytogenes*. The cDNA is comprised of an open reading frame (ORF) of 990 bp and encodes a polypeptide of 329 residues. TmPGRP-LE contains one PGRP domain, but lacks critical residues for amidase activity. Quantitative RT-PCR analysis showed a broad constitutive expression of the transcript at various stages of development spanning from larva to adult. RNAi mediated knockdown of the transcripts, followed by a challenge with *L. monocytogenes*, showed a significant reduction in survival rate of the larvae, suggesting a putative role of TmPGRP-LE in sensing and control of *L. monocytogenes* infection in *T. molitor*. These results implicate PGRP-LE as a defense protein necessary for survival of *T. molitor* against infection by *L. monocytogenes*.

## 1. Introduction

Innate immunity serves as a major line of defense in insects and recognizes, modulates and signals effector functions against invading pathogens. The first important step in innate immunity is the recognition of pathogen-associated molecular patterns (PAMPs), which are exclusively found in microbes as dangerous, non-self entities. This role is accomplished through the activity of various molecular families capable of sensing the presence of invading microbial pathogens in either hemolymph or various immune cells (hemocytes, fat cells, gut epithelial cells) of host organisms [1]. Such molecular families, collectively called pattern recognition receptors (PRRs), include the toll-like receptors in mammals, and β-1,3-glucan recognition proteins (βGRPs), Gram-negative binding proteins (GNBPs) and peptidoglycan recognition proteins (PGRPs) in insects and other invertebrates.

Peptidoglycan (PGN), a PAMP in the cell walls of both Gram-positive and Gram-negative bacteria, consists of *N*-acetylglucosamine and *N*-acetylmuramic acid in β-1,4 linkages, cross-linked by short peptide stems composed of alternating l- and d-amino acids [2]. The PGRP family of PRRs is an integral component of host innate immune response as its members are required for the recognition of PGNs and for subsequent activation of antimicrobial peptides (AMPs) [3]. PGRPs were first characterized in the moths, *Bombyx mori* and *Trichoplusia ni* [4,5], and have since been identified in insects, mollusks, echinoderms and vertebrates [3,6,7], but not in plants and nematodes [3]. In insects, up to 19 different PGRPs have so far been identified [3]. In *Drosophila*, members of the PGRP family have been found in the hemolymph and on the surface and/or inside immune competent cells [8,9]. Some of these members have been classified as catalytic PGRPs, including PGRP-SC1a, SC1b and PGRP-LB, which cleave PGN between *N*-acetylmuramic acid of the backbone and l-alanine in the stem peptide [10,11]. Others are the non-catalytic forms including PGRP-SA, PGRP-LE, PGRP-LC and PGRP-SD and constitute the majority of known PGRPs. These can bind to PGNs leading to the activation of the Toll and Imd signal transduction pathways. These pathways eventually control the production AMPs through either nuclear factor kappa-light-chain-enhancer of activated B cell (NF-κB) or Relish transcription factors [9].

Although most PGRPs function as extracellular microbial sensors, PGRP-LE has been shown to play a role in intracellular bacterial recognition in addition to its extracellular roles [12]. A previous report [9] showed that PGRP-LE is responsible for recognition of an intracellular DAP-type PGN containing the bacteria, *Listeria monocytogenes* and subsequently inducing autophagy to inhibit the pathogen’s growth in the cytosol of *Drosophila* immune cells [9]. PGRP-LE is known to be present in both the hemolymph and inside immune cells and specifically binds to the *meso*-diaminopimelic acid (DAP)-type PGN. In the hemolymph, PGRP-LE preferentially activates Imd signaling pathway in a non-cell autonomous manner and depends mainly on the activity of another member of the family, PGRP-LC [11]. Such a synergistic action of PGRP-LE and PGRP-LC has been reported in *Drosophila*, wherein the single mutants (*PGRP-LE^112^* and *PGRP-LC^7454^*) showed complete resistance but the double mutants (*PGRP-LE^112^*/*PGRP-LC^7454^*) were found susceptible against *E. coli*. Also, while *PGRP-LC* mutant *Drosophila* flies had highly reduced expression of AMPs after infection with *E. coli* and other Gram-negative bacteria such as *Erwinia carotovora*, *PGRP-LE* mutants showed normal expression of transcripts [13].

In the mealworm beetle, *Tenebrio molitor* (Coleoptera: Tenebrionidae), roles of individual PGRP family members, such as PGRP-SA [14] and PGRP-SC2 [15] have been reported. TmPGRP-SA acts by binding to Lys-type PGN leading to the recruitment of GNBP1 and modular serine proteases to form a complex which acts as an initial activator triggering serine protease cascades in the toll and proPO pathways in response to infections [14]. Similarly, TmPGRP-SC was confirmed as specifically induced by injection of monomeric DAP-type and polymeric DAP- and Lys-type PGN into *Tenebrio* larvae. Moreover, TmPGRP-SC2 showed strong *N*-acetylmuramic-l-alanine amidase activity against DAP-type PGN and only a weak activity against Lys-type PGN [15]. There are no reports on the existence of PGRP-LE in *T. molitor* so far, that acts as a main microbial sensor of Imd signal transduction pathway in insects. We report the identification and partial characterization of a novel PGRP-LE homologue in *T. molitor* and show through RNAi that *TmPGRP-LE* contributes to the host’s ability to control and survive against *L. monocytogenes* infection. It must be noted here that although *L. monocytogenes* is not a natural pathogen of insects, it is generally accepted as a convenient tool for addressing innate immune and antibacterial defense of insect hosts.

## 2. Results and Discussion

### 2.1. Characterization of *TmPGRP-LE* Full-Length cDNA

**Figure 1:**
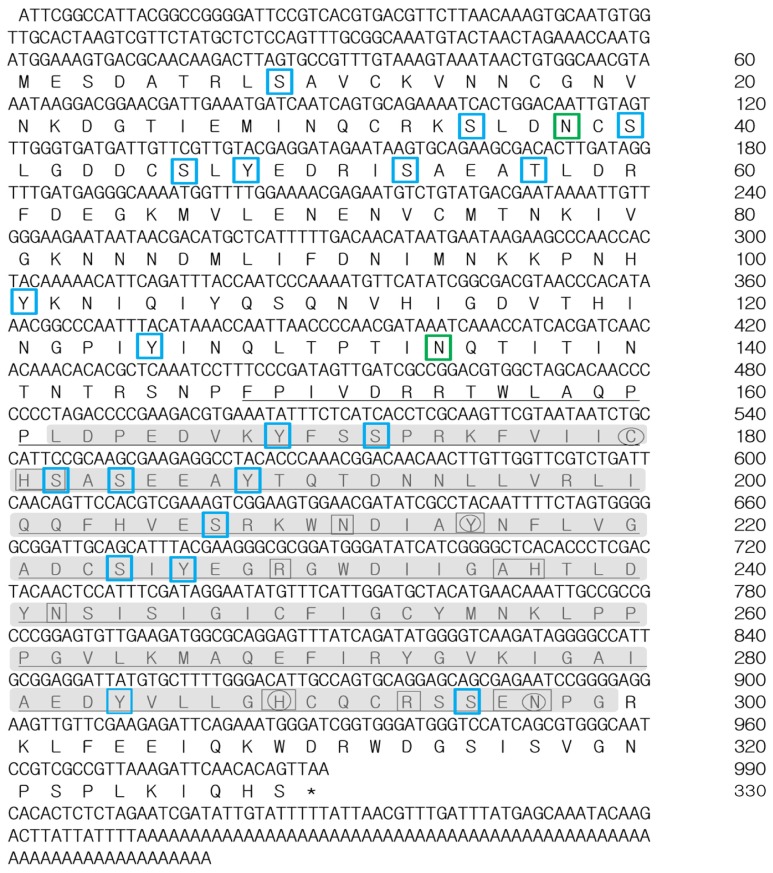
Nucleotide and deduced amino acid sequence of a novel homologue of PGRP-LE from *T. molitor*. The nucleotides and amino acids are numbered along the right margin beginning with the translation start (ATG) and ending with the stop codon (TAA) highlighted with an asterisk. The amidase and PGRP domains have been shaded and underlined respectively. The predicted *N*-glycosylation sites have been shown in green boxes, whereas the predicted serine, threonine and tyrosine phosphorylation targets have been shown in blue boxes. The conserved zinc binding residues have been encircled. The predicted PGN recognition residues have been boxed.

A single expressed sequence tag (EST) homologous to known and fully characterized PGRPs of other organisms was identified from the sequencing of random clones of *T. molitor* cDNA library. Re-sequencing of the identified EST yielded a full-length sequence comprised of 1248 nucleotides (Figure 1). The *TmPGRP-LE* open reading frame (ORF) is comprised of 990 nucleotides encoding a polypeptide of 329 amino acids with a predicted molecular weight of 37.3 kDa. The 5′-, 3′-non-coding and poly (A^+^) sequences were 119, 72 and 67 bp, respectively. The signal peptide prediction for TmPGRP-LE was negative, suggesting its existence in the cytoplasm, where it acts as an intracellular receptor for DAP-type PGN [11]. Intriguingly, the extracellular roles of PGRP-LE upstream of and in coordination with PGRP-LC to recognize PGN and activate the Imd pathway have been reported [13,16]. However, the lack of signal peptide in TmPGRP-LE is consistent with the observation that other long-form members of the family (*i.e.*, PGRP-LA to PGRP-LF) possess no signal peptide [17,18], even though PGRP-LA, LC and LD have a transmembrane region, encoding membrane proteins. Like PGRP-LE, PGRP-LB, which is also not a transmembrane protein, is reported to lack an export signal and probably remains in the cytoplasm as an intracellular PGN scavenger [18].

The putative glycosylation sites in TmPGRP-LE have been predicted to asparagine at position 39 and 135 having glycosylation potential of 0.6886 and 0.6038, respectively, which is well above the threshold of 0.5. But as TmPGRP-LE lacks a putative signal peptide, it is highly unlikely that it would be exposed to the *N*-glycosylation machinery and thus may not be glycosylated.

Putative phosphorylation sites in deduced amino acid sequence of TmPGRP-LE were predicted to be serine at positions 10, 36, 41, 47, 54, 172, 183, 185, 208, 225 and 296, threonine at position 58 and tyrosine at position 49, 102, 126, 170, 189, 227 and 285 [19]. The prediction for *N*-acetylation was positive, as there was a serine at position 3 in the amino acid sequence of TmPGRP-LE. The theoretical pI of the protein was 5.97 (charge at pH 7.0 = −5.099), and the molecular mass was 37,320.42 Da as predicted with Protean software analysis program of Lasergene suite. Apart from the above, the compositional analysis of TmPGRP-LE included 33 strongly basic (10.03%), 39 strongly acidic (11.85%), 100 hydrophobic (30.40%) and 106 polar (32.22%) amino acid residues with the charged residue count of 104 (31.61%) [20]. In case of *Drosophila* PGRP-LE, TmPGRP-LE also showed a highly charged *N*-terminal domain connected to the PGRP domain. The computed extinction coefficient of the protein was 46,130 M^−1^·cm^−1^, in case all the cysteines in the sequence form cystines and 45,380 M^−1^·cm^−1^, in case all the cysteines get reduced. The computational values should be considered reliable as TmPGRP-LE contains tryptophan residues. Normally, proteins without tryptophan residues can form a 10% error in the extinction coefficient predictions. The instability index for the protein was 38.65, which classified the protein as stable, whereas the aliphatic index was 83.83. Also, the general average of hydropathicity (GRAVY) plot, calculated as the sum of hydropathy values of all amino acid residues in the sequence, was −0.416 [21], suggesting its elevated hydrophilic properties.

To understand the homology of the novel gene product from *T. molitor* genome, we conducted BLAST analysis with the deduced amino acid sequence (results not shown). The sequences found were used to search for additional members of the PGRP gene family. TmPGRP-LE sequence showed highest similarity of 65% with that of its close relative, *Tribolium castaneum* PGRP-LE (TcPGRP-LE), which followed with a low homology of about 38% with a non-relative companion, *Armigeres subalbatus* PGRP-LE (AsPGRP-LE).

Furthermore, the analysis included the majority of known PGRP homologues and their isoforms from *D. melanogaster*, grouped into two major classes of short (including PGRP-SA, SB1and SB2, SC1A and SC1B, SC2 and SD) and long (PGRP-LA, LB, LC, LD, LE and LF) forms. The classes of PGRP homologues, their distribution and diversification within the *Drosophila* genome have been reported [22]. TmPGRP-LE showed a homology of about 36% with *D. melanogaster* PGRP-LE-A and PGRP-LE-B isoforms (DmPGRP-LE-A and DmPGRP-LE-B), as well as DmPGRP-LB (isoforms A, B, E and F), and a homology of about 33% with DmPGRP-LF and other DmPGRP-LB isoforms such as A, C and D. The amino acid sequence homology of TmPGRP-LE with shorter forms of *Drosophila* PGRPs was evaluated to be in the range of 30%–34% with maximum relatedness to DmPGRP-SB2. The multiple sequence alignment with the percentage identity analysis showed a significant variability in the number and pattern of *N*-terminal amino acids within the PGRPs characterized from insects thus far. This reflects the categorization of the family into PGRP-S, that are usually small (about 20 kDa) and predominantly secreted extracellular proteins, intermediate forms (PGRP-I) that are 40–45 kDa transmembrane proteins and PGRP-Ls up to 90 kDa, which may be intracellular, transmembrane, or extracellular proteins [3].

**Figure 2:**
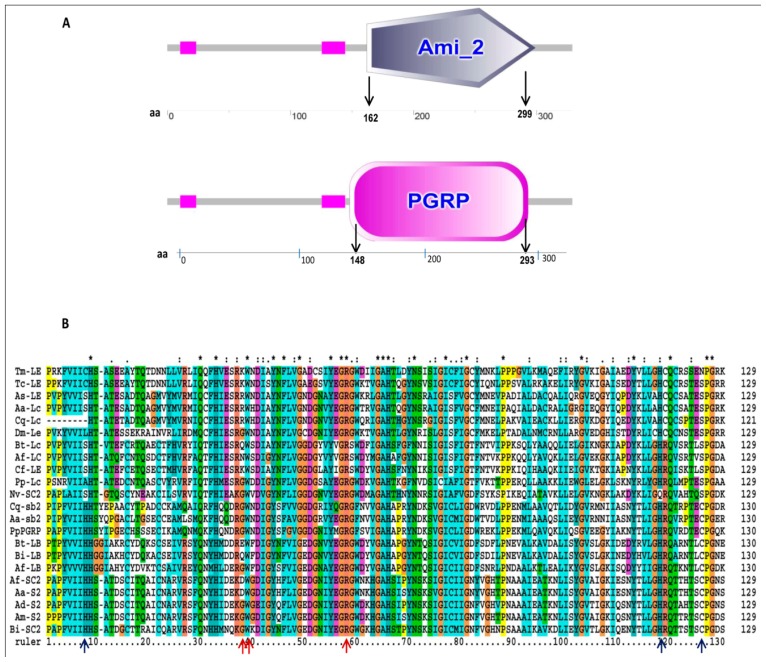
Characterization and alignment of amino acid sequences of TmPGRP-LE homologues. (**A**) Domain architecture predicted using SMART analysis tool. Ami-2 and PGRP stand for predicted amidase-2 and PGRP domains, respectively; and (**B**) Multiple sequence alignment of amidase/PGRP domains of PGRP family members in representative insects. The sequences have been compiled from NCBI database and have been represented by accession numbers (Figure 3 for details). Catalytic residues for amidase activity and specific PGN recognition residues are indicated by blue and red arrows, respectively. Residues shared are indicated with the following symbols: “*” (denotes identical residues in all sequences); “:” (conserved substitutions according to similar properties of amino acids); “.” (indicates semi-conserved amino acid substitutions). Dashes indicate gaps used to maximize the alignment.

We further dissected the TmPGRP-LE sequence by the identification of PGRP domain signature sequence using the SMART analysis program (Figure 2A). As expected, the highly conserved PGRP domain spanning from amino acid residue 148 to 293 (*E*-value 4.95 × 10^−59^), was found overlapping with the predicted amidase domain spanning from amino acid number 162 to 299 (E-value 2.97 × 10^−11^) at the *C*-terminus. All PGRPs share a highly conserved PGRP domain (approximately 160 amino acids on their *C*-termini) with similarities to the zinc-dependent *N*-acetylmuramoyl-l-alanine amidase of bacteriophage T7 lysozyme [11,18,23–25]. Therefore we found it appropriate to discuss the predicted PGRP domain of TmPGRP-LE in comparison with similar sequences from other representative insects. In order to predict the catalytic and specific PGN recognition residues in TmPGRP-LE, we conducted a detailed analysis of the 129 amino acid residues in the PGRP domain (from amino acid 173 to 293). For the same, we made a comparison of the domain with similar domains of PGRP-LE families in insects by sequence alignment, percentage identity matrix, and subsequent phylogenetic tree analysis. The alignment of PGRP domain of TmPGRP-LE was useful towards examining the amino acid residues that form the Zn^2+^ binding site, responsible for catalytic (amidase) mode of action (Figure 2B). Structural studies have highlighted that the zinc binding activities in PGRPs are primarily coordinated with the help of two histidines in the overlapping domain and a cysteine in the binding groove of PGRP domain. Although the residues are strictly conserved in PGRPs (especially in fish and humans), the non-catalytic PGRPs lack the potentiality of binding to Zn^2+^.

In the case of TmPGRP-LE, the lack of a histidine and a cysteine at position 8 and 126 of the amidase/PGRP domains may be relevant in explaining its non-catalytic (non-amidase) activity on PGN [26]. The position of amino acid 297 is always associated with a cysteine with amidase activity in PGRPs and most often is occupied by serine in PGRPs devoid of amidase activity. *T. castaneum* PGRP-LB (TcPGRP-LB) and PGRP-SB (TcPGRP-SB) also shows to contain key residues for an amidase activity [27]. Similarly, in humans, PGLYRP-2 possesses *N*-acetylmuramoyl-l-alanine amidase activity while PGLYRP-1, PGLYRP-3 and PGLYRP-4 do not [23,25].

The retention in conservation of catalytic residues related to amidase activity has also been a feature among the oyster *Crassostrea gigas* PGRP (CgPGRP) proteins [28]. In addition, the amidase activity was significant in both cases of full protein and also in the form of isolated amidase domain in recombinant *Sciaenops ocellatus* PGRP homologue (rSoPGLYRP-2), suggesting that the amidase domain may be able to recognize the PAMPs and process PGN lysis [29]. The close spacing of amino acids involved in amidase activity and PGN recognition may suggest the formation of two distinct active centers. Also, the three residues (39G, 40W, 61R) involved in PGN recognition [30] are highly conserved with a certain degree of mutation, indicating their critical function and also the evolutionary pressure to serve in the capacity of PGN recognition. Significantly, it has to be noted here that, PGN structures from different bacteria present a remarkable set of variability in their peptide stems, with certain degree of cross-linking adding to variability [30]. PGN recognition sites are thus expected to vary accordingly. In TmPGRP-LE, and its close counterpart TcPGRP-LE, the substitution (39G to K) may indicate that affinity and/or specificity to PGN might differ from other members of the family.

### 2.2. Phylogenetic Analysis of *TmPGRP-LE*

**Figure 3:**
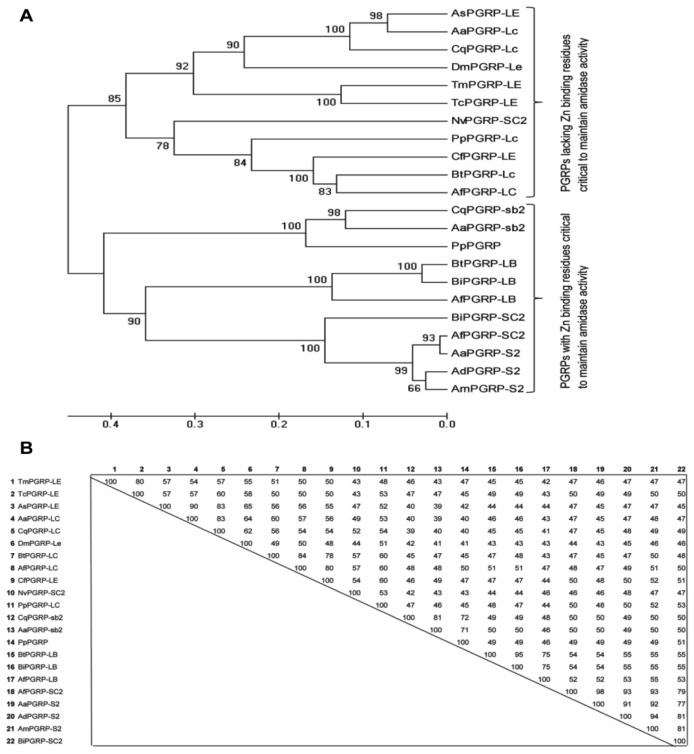
Molecular phylogenetic and percentage identity analysis of TmPGRP-LE (**A**) Phylogenetic analysis as performed by MEGA 5.0 software with representative PGRP gene family members. The percentage of replicate trees in which the associated taxa clustered together in bootstrap test (5000 replicates) is shown on interior branches. The abbreviations and accession numbers of the sequences were as follows: PGRP-LE: *Tribolium castaneum* (TcPGRP-LE; XP_968926.1), *Armigeres subalbatus (*AsPGRP-LE; AEX31482.1), *Drosophila melanogaster* (DmPGRP-LEa; NP_573078.1), *Camponotus floridanus* (CfPGRP-LE; EFN63542.1); PGRP-LC: *Aedes aegypti (*AaPGRP-LC; XP_001656352.1), *Culex quinquefasciatus* (*CqPGRP-LC*; XP_001842237.1), *Bombus terrestris* (BtPGRP-LC; XP_003396511.1), *Apis florea* (AfPGRP-LC; XP_003693124.1); *PGRP-SC2: Bombus impatiens* (XP_003396511.1), *Nasonia vitripennis (*NvPGRP-SC2; XP_001603488.1), *Apis florea* (AfPGRP-SC2; XP_003694493.1); PGRP-SB2: *Culex quinquefasciatus* (CqPGRP-SB2; XP_001849091.1), *Aedes aegypti (*AaPGRP-SB2; XP_001654275.1); PGRP-LB: *Bombus terrestris* (BtPGRP-LB; XP_003400160.1), *Bombus impatiens* (BiPGRP-LB; XP_003493260.1), *Apis florea (*AfPGRP-LB; XP_003694545.1); PGRP-S2: *Apis mellifera* (AmPGRP-S2; NP_001157188.1), *Apis dorsata* (AdPGRP-S2; ACT66892.1); *Phlebotomus papatasi (*PpPGRP; ABV60369.1); and (**B**) Percentage identity matrix of the representative species as inferred using ClustalX 1.83.

We made an attempt to deduce the phylogenetic relationship of TmPGRP-LE with the PGRP domains of other representative members. For the same, we constructed a distance tree using the unweighted pair group method with arithmetic mean (UPGMA). The consensus of the tree was inferred from 5000 bootstrap replications, with most relationships found to be uncertain. This suggested that the PGRP family is highly diversified (Figure 3A) with two major groups identified based on the amidase activity. TmPGRP-LE was found classified into the PGRP class lacking Zn binding residues and was placed together with TcPGRP-LE. The genes within the PGRP-SB and SC clusters were closely related, and most significantly, there was a tendency for long and short forms to form separate monophyletic groups.

The separation of short and long forms of PGRPs is understandable as there happens to be separated evolutionary histories with all the short PGRP genes derived from an ancestral pattern with two introns and subsequently losing out on one or both of introns during the course of evolution. The introns in long PGRPs do not correspond to introns of short genes as previously shown in *Drosophila* [22]. Notwithstanding the same, PGRP-LB was found to be an exception, and seems to cluster with the short or the long forms depending on the sequences used for comparison.

In addition, the percentage identity matrix of the selected PGRP domain showed greater levels of homology as compared with the full-length cDNA (Figure 3B). This was not surprising as the full-length sequence contains an *N*-terminus that is greatly unstable. The identities among the sequences increased appreciably due to the conserved status of the domains, and were about 80% with TcPGRP-LE and about 55% with DmPGRP-LE-A. Interestingly, the homology of TmPGRP-LE with PGRPs from mosquitoes such as *Armigeres subalbatus* PGRP-LE (AsPGRP-LE), *Aedes aegypti* PGRP-LC (AaPGRP-LC) and *Culex quinquefasciatus* PGRP-LC (CqPGRP-LC) was found to be in the range of 54%–57%. This conforms to the earlier report on beetle and mosquito *PGRP-LA* genes encoding two alternative splice forms and *PGRP-LA* and –*LC* genes placed next to each other in the same cluster [27].

### 2.3. Secondary Structure and Homology Modeling of *TmPGRP-LE*

The secondary structure prediction of TmPGRP-LE was done using PSIPRED software, and it showed to be composed of sheet, coil, and helical regions (Figure S1A). The inconsistency in the *N*-terminal region is evident, as most of the structure takes the form of coils with interspersed sheet and helix regions. The tertiary structure of amidase/PGRP domain was predicted with significant authority (Figure S1B). TmPGRP-LE amidase/PGRP domain model showed the presence of a central β-sheet with five β-strands and three α-helices. A previous report has suggested that amino acid residues subject to positive selection are basically located at the periphery of coils and α-helices rather than in central β-sheet or active center, a finding that is consistent with the insect PGRP family [31]. The Swiss-model prediction for the PGRP domain of PGRP-LE was based on homology modeling using *Drosophila* PGRP-LE in complex with tracheal cytotoxin as a template (PDB: 2cb3) with high quality [32].

The estimated model (Figure S1C) reliability, reflected in the QMEAN4 score of 0.62, was appropriate. Furthermore, the atomic non-local environment assessment (ANOLEA) value computed was >0, suggesting an accurate model prediction for the conserved domain [33].

### 2.4. Temporal Expression Patterns of *TmPGRP-LE*

**Figure 4:**
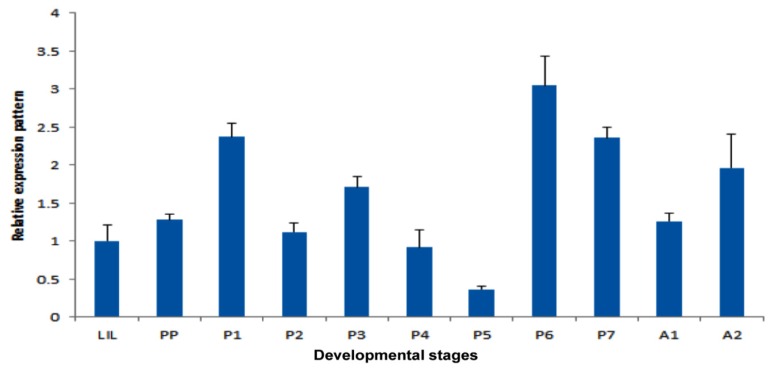
Temporal expression patterns of *TmPGRP-LE. TmPGRP-LE* transcripts at various developmental stages were quantified by real time PCR. Expression levels of the target gene were normalized to the expression of *T. molitor* ribosomal protein *L27a* (*TmL27a*) gene as an internal control and compared to that of the last instar larva. Statistical bars indicate mean ± SEM for three independent experiments.

We analyzed the expression patterns of *TmPGRP-LE* gene, during various developmental stages (late larval, pupal and adult stages) of *T. molitor* by quantitative PCR method. The real-time transcript profile showed a constitutive expression of the gene at all stages of growth with lowest transcript levels observed at pupal stage (day 5) (Figure 4). This observation is in agreement with previous reports [12,22] suggesting that long PGRPs are constitutively expressed in insects with an exception of *PGRP-LB*, which is inducible upon immune challenge.

### 2.5. RNAi-Mediated Knockdown of *TmPGRP-LE* Leads to Reduced Survival Rate of *T. molitor* Larvae infected with *L. monocytogenes*

**Figure 5:**
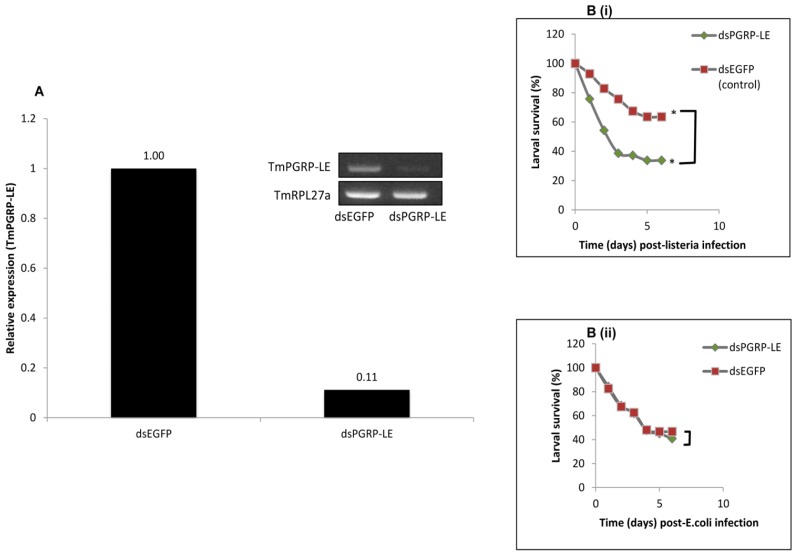
Effect of *TmPGRP-LE* knockdown on the survivability of *T. molitor* larvae (**A**) Silencing of *TmPGRP-LE* mRNA in the larvae of *T. molitor* by RNA interference. Real time qRT-PCR and semi-quantitative PCR methods were used to study the gene knockdown levels in the group injected with *dsPGRP-LE* prior to immune challenge by *Listeria*. The control group was injected with *dsEGFP*. A housekeeping gene *TmRPL27a* was used as an internal control during PCR reactions; (**B**) Survival pattern of *T. molitor* larvae following immune challenge by *L. monocytogenes*. Larvae whose gene expression was down regulated by RNAi exhibited a significant (*p* < 0.05) (Wilcoxon-Mann-Whitney test) reduction in survivability as compared to the control group “*” represent a statistically significant difference between means (**B**-**i**); Replacing *L. monocytogenes* with *E. coli* did not result in the differential survival pattern observed with the intracellular pathogen *L. monocytogenes* (**B**-**ii**). Results presented here are an average of three independent biological replicate experiments.

PGRP-LE is known to recognize DAP-type PGN containing bacterial intruders into the host system and preferentially activate the Imd pathway. It was therefore hypothesized that down-regulation of *TmPGRP-LE* by RNAi would (i) impact the expression levels AMPs and (ii) compromise the host’s ability to recognize and control intracellular *L. monocytogenes*. To this end, we first knocked down *TmPGRP-LE* mRNA transcripts by injecting *dsPGRP-LE* to the LIL as described in section 3.10. As a control group, the LIL were injected with double-strand enhanced green fluorescent protein (*dsEGFP*). Following successful knockdown of *TmPGRP-LE* transcripts by RNAi (Figure 5A), the expression pattern of AMP genes such as cecropin, coleoptericin, defensin, lysozyme, tenecin1, tenecin 2 and tenecin 3 was monitored before and after challenge with DAP-type PGN-containing bacteria, *E. coli* and *L. monocytogenes*. The knockdown of *TmPGRP-LE* was unable to bring any significant change in the expression levels of the AMPs with or without bacterial challenge (data not shown). This could be partly due to a previously reported redundancy between PGRP-LE and -LC in sensing DAP-type PGN and subsequent activation of Imd pathway. In a study, *PGRP-LE^112^/PGRP-LC7^454^* double mutants were found to be more susceptible to *E. coli* infections than either of the mutants alone [13]. Furthermore, PGRP-LE has been shown to be dispensable *in vivo*, for systemic sensing of the monomeric form of PGN (the tracheal cytotoxin, TCT) and the activation of Imd pathway following infections by DAP-type PGN containing bacteria such as *E. coli* and *Erwinia carotovora* 15 [13,34]. Taken together, we believe that TmPGRP-LE plays an indispensable role in sensing the extracellular PGNs and that it may putatively activate Imd pathway upon infection by DAP-type peptidoglycan, more so under conditions where *PGRP-LC* expression remains unaltered.

The suggestions that the cytosolic recognition of the monomeric form of DAP-type PGN (TCT) by PGRP-LE, is neither dispensable nor PGRP-LC-dependent [8,34], led us to hypothesize that TmPGRP-LE may play a role in the control of *L. monocytogenes* infection in the host, thus improving its survivability. To examine the effects of *TmPGRP-LE* RNAi on the ability of *T. molitor* LIL to survive *E. coli* and *L. monocytogenes* infections, we performed a larval survival assay. An initial study using saline (0.9% NaCl) alone to challenge *TmPGRP-LE* RNAi larvae showed no death in all controls and treatment groups, including the *dsEGFP* injection group (data not shown in figures). Subsequently, we focused on challenging *TmPGRP-LE* RNAi larvae with *E. coli* and *L. monocytogenes*. Five days post-injection of dsRNAs, 2 μL (~1000 CFU) of an overnight culture of *L. monocytogenes* (in saline) were injected per larva. The survival assay with the bacterial challenges was repeated three times using three independent cohorts of larvae, (*n* = 30) in each biological replicate. Rates of survival between the *TmPGRP-LE* RNAi and *dsEGFP*-injected larvae were scored daily for six consecutive days. The survival experiment was repeated with *E.coli*, a DAP-type PGN-containing bacterium that does not survive in phagocytic cells.

The survival data revealed a differential rate of survival between *dsPGRP-LE* and *dsEGFP* injected larvae when challenged with *L. monocytogenes*. The *PGRP-LE* knockdown group showed a significantly (*p* < 0.05) reduced survival rate when compared with the control group (Figure 5B-i). This difference in survival rate was not observed when *L. monocytogenes*, was replaced by *E. coli* (Figure 5B-ii). The survival curves for both the knockdown and control group were similar in the case of *E. coli* treatment. This indicates that neither groups showed superiority in the resistance against *E. coli* infections. The finding that *TmPGRP-LE* RNAi did not alter the survival of *T. molitor* larvae after *E. coli* challenge is in general agreement to a previous report [35] which found that *PGRP-LE* RNAi did not affect the survival rates of a mosquito *Armigeres sabalbatus*, after challenge by *E. coli* and *Micrococcus luteus*, despite the fact that the knockdown caused significant reductions in the transcription of two AMP genes, cecropin A and defensin C1. In *Drosophila*, intracellular PGRP-LE has been shown to be responsible for detecting the invading *L. monocytogenes* and subsequently inducing its autophagic control [9]. Our data showed that *TmPGRP-LE* RNAi makes *T. molitor* very susceptible to *L. monocytogenes* infections, suggesting that TmPGRP-LE is also involved in the recognition and/or immune responses to DAP-type PGN containing bacteria.

## 3. Experimental Section

### 3.1. Insect Collection and Maintenance

*T. molitor* larvae were procured from College of Pharmacy, Pusan National University, Busan, South Korea and maintained on wheat bran meal in an environmental chamber at 26 ± 1 °C with 60% ± 5% relative humidity and a 16:8 h light and dark cycle. Only last instar larvae were used for all experiments unless otherwise stated. To ensure uniformity in size, the larvae were separated according to their physical sizes using a set of laboratory test sieves (Pascall Eng. Co. Ltd, Crawley, Sussex, UK). Only healthy larvae that passed through a sieve of 2.80 mm aperture size and retained by the 2.00 mm aperture size sieve were selected for experiments.

### 3.2. Chemicals

All chemicals used for the experiments were of analytical grade, obtained from Sigma Chemical Co. (St. Louis, MO, USA) unless otherwise mentioned in the text.

### 3.3. Bacterial Strains and Media

*L. monocytogenes* strain ATCC 7644 used in the study was directly procured from the American Type Culture Collection (ATCC) and the *E. coli* ATCC 25922 strain was a kind gift from Prof. Oh, S.-J., Division of Animal Science, Chonnam National University, Gwangju, South Korea. The media used for the multiplication and colony forming unit (CFU) determination of the bacteria were (i) Luria Bertani (LB) [Tryptone 10 g, Yeast extract 5 g, Sodium chloride 10 g (pH, 7.0) per liter] and (ii) Brain-heart infusion (BHI) for *E. coli* and *L. monocytogenes*, respectively.

### 3.4. Construction of Full-Length Enriched cDNA Library of *T. molitor* Larvae

Total RNA from *T. molitor* larvae (*n* = 200) was isolated by TRIzol reagent (Molecular Research Centre, Inc., Cincinnati, OH, USA) after homogenization using TissueLyser (Qiagen, Valencia, CA, USA) and subsequently mRNA was purified using Absolutely mRNA Purification Kit (Stratagene, Santa Clara, CA, USA). The cDNA library was synthesized using Express cDNA Synthesis Kit (Stratagene, Santa Clara, CA, USA). The cDNAs of more than 500 bp in length were ligated into pBK-CMV vector (Agilent Technologies, Inc., Santa Clara, CA, USA) and packaged using the ZAP expression cDNA Gigapack^®^ III Gold cloning kit (Stratagene, Santa Clara, CA, USA) according to manufacturer’s instructions. cDNA library clones (~2000) were cultured in Terrific broth (TB) medium containing kanamycin (50 mg/mL) at 37 °C for 15 h. Plasmid DNAs extracted from the selected colonies were sequenced by ABI 3730 XL capillary sequencer (Applied Biosystems, Foster City, CA, USA). Clones corresponding to the partial sequence of PGRP-LE (TmPGRP-LE) were identified, by conducting BLASTX with Swissprot analysis (EMBL-EBI, Hinxton, Cambridge, UK). By using the plasmid DNA as templates, full-length sequencing was performed by a clone-by-clone primer walking method using a Model 3730 XL sequence analyzer (Applied Biosystems, Foster City, CA, USA). Sequencing reaction was performed using the BigDye Terminator version 3.1 cycle sequencing kit (Applied Biosystems, Foster City, CA, USA). Sequences were assembled using the Phrap program (University of Washington, Seattle, WA, USA) [36]. Based on the assembled sequences, primers corresponding to the terminal sequences were designed using Primer3 (version 0.4.0, Whitehead Institute, Cambridge, MA, USA) [37]. The primer walking procedure was repeated until poly (A) tail region for the sequence was identified. The full-length sequences were finished by trimming the vector-derived sequences from both ends using the cross match program. The consensus full-length cDNA sequence of TmPGRP-LE obtained from 200 *T. molitor* larvae has been registered with the European Nucleotide Archive-European Bioinformatics Institute (ENA-EBI, Hinxton, Cambridge, UK) with the accession number HF935084.

### 3.5. *TmPGRP-LE* Sequence Analysis

The cDNA and deduced amino acid sequence of *TmPGRP-LE* was analyzed using UltraEdit-32 Professional Text/HEX editor (version 12.00, IDM Computer Solutions Inc., Hamilton, OH, USA) software package and deduced amino acid sequence was predicted by Open Reading Frame finder at National Centre for Biotechnology Information (NCBI) [38] and the Expert Protein Analysis System (Swiss Institute of Bioinformatics, Lausanne, Switzerland) at ExPASy bioinformatics resource portal [39]. The conserved protein domains were identified by using the Simple Modular Architecture Research Tool (SMART) (version 4.0, EMBL, Heidelberg, Germany). Multiple sequence analysis and percentage identity matrix of the conserved amidase/PGRP domains in the PGRP-LE homologue from *T. molitor* was done in comparison with other representative PGRP-LEs from insects using Clustal X (version 2.0.12, University of Strasbourg, Strasbourg, France) [14]. The PGRP-LE protein sequences of representative insect groups were extracted from the GenBank repository at NCBI and have been presented in Table 1. The prediction of the putative signal peptide sequence was done at the Signal 4.0 server (www.cbs.dtu.dk) [40]. The protein sequence analysis tools used in the study towards the prediction of theoretical *M*_W_ and isoelectric point (pI) was done at the ExPASy bioinformatics resource portal (http://expasy.org) [39]. ProtParam [41] at ExPASy bioinformatics resource portal was used to compute the various physical and chemical parameters of the deduced protein sequence. PeptideCutter [42] at ExPASy bioinformatics resource portal was used to predict the potential proteases cleavage sites in the sequence. ProtScale [43] at ExPASy bioinformatics resource portal was used to compute and represent the profile produced by amino acid scale on the protein. The prediction of *N*-glycosylation sites was confirmed at NetNGlyc 1.0 server (Technical University of Denmark, Lyngby, Denmark). Post-translational modifications such as *N*-acetylation, *O*-glycosylation, phosphorylation and kinase-specific phosphorylation were predicted with the aid of NetAcet 1.0, NetOGlyc, NetPhos 2.0 and NetPhosK 1.0 server respectively (Technical University of Denmark, Lyngby, Denmark). The potential coding region was predicted using FGENESH (Softberry, Inc., Mount Kisco, NY, USA) [44]. ORF and protein statistics were inferred by the EditSeq tool of Lasergene 9.0 software (DNASTAR Inc., Madison, WI, USA). The software was also used to study the codon usage, base composition in the ORF and predicted structural class of the protein.

### 3.6. Phylogenetic Analysis

Prior to phylogenetic analysis, Clustal X software (version 2.012, University of Strasbourg, Strasbourg, France) was used to perform multiple sequence alignment of the conserved amidase/PGRP domains from the deduced amino acid sequence of novel PGRP-LE homologue in *T. molitor* in comparison with related conserved domain sequences of PGRPs in other representative insects. MEGA 5.0 [45] software (The Biodesign Institute, Tempe, AZ, USA) was used to construct the consensus phylogenetic tree using the UPGMA method [46]. The evolutionary distances for the representative sequences were computed using the Poisson correction and are in the units of the number of amino acid substitutions per site. The bootstrap values were estimated by 5000 replications.

### 3.7. Secondary Structure Prediction and Modeling

Secondary structure predictions of the conserved PGRP domain were performed using the consensus prediction program available at PSIPRED protein structure prediction server 2.6 (Bloomsbury Centre for Bioinformatics, London, UK) [47]. The generated consensus leads to three possible states for each residue (“H”: alpha helix, “E”: Extended strand and “C”: Coil). The accuracy of prediction may reach a score of 80.7%. Three-dimensional structures were modeled using SWISS-MODEL, a fully automated protein structure homology modeling server (http://swissmodel.expasy.org) [48] and PGRP-LE in complex with tracheal cytotoxin (monomeric DAP-type PGN) (PDB code: 2cb3) was used as the template. Model quality was evaluated by Anolea and QMEAN [49], the structure of the model was visualized by using PyMol molecular graphics system (version 1.5, Schrodinger, Cambridge, MA, USA).

### 3.8. Developmental Expression Pattern of *TmPGRP-LE*

For the expression patterns of *TmPGRP-LE*, total RNA was isolated from the whole body (*n* = 5) at various stages of development in *T. molitor*, namely, last instar larva (LIL), pharate pupa (PP), pupa day 1 to 7 (P1–P7) and adult day 1 and 2 (A1–2) using SV Total RNA isolation system (Promega Corporation, Madison, WI, USA) by following manufacturer’s instructions. From the isolated total RNA, cDNA corresponding to each stage of growth was synthesized using AccuPowerR RT PreMix kit (Bioneer, Daejeon, Korea) by following the instructions in the kit. The synthesized cDNAs were used as templates for the quantitative PCR (qPCR) reactions performed on an Exicycler™ 96 real-time quantitative thermal block (Bioneer, Korea) using primers (Table 1) at an initial denaturation of 95 °C for 20 s, followed by 45 cycles of denaturation at 95 °C for 5 s and annealing at 60 °C for 20 s. The 2^−ΔΔ^*^Ct^* method was employed to analyze the expression levels of *TmPGRP-LE. TmRPL27a* gene was used as an internal control to normalize the expression [50–53]. Specificity of qPCR primers was confirmed by gel electrophoresis.

### 3.9. RNA Interference of *TmPGRP-LE*

The PCR product containing a specific region of *TmPGRP-LE* gene was used to generate a template for *in vitro* transcription using gene-specific primers tailed with a T7 promoter sequence (Table 1). To minimize potential off-target effects, primers were designed using the SnapDragon dsRNA design software (http://www.flyrnai.org/cgi-bin/RNAi_find_primers.pl) [54], which detected no potential off-target regions in the sequence [55,56]. The *dsRNA* was prepared with the Ampliscribe™ T7-Flash™ transcription kit (Epicentre Biotechnologies, Madison, WI, USA) according to manufacturer’s instructions. The synthesized dsRNA was purified by ammonium acetate precipitation as described in the protocol attached to the kit. The integrity of the purified dsRNA was checked by running 1% agarose gel for 20 min at 100 V and quantified using NanoDrop spectrophotometer (Thermo Scientific, Wilmington, DE, USA). Purified dsRNA was stored at −20 °C until injected into *T. molitor* larvae. Cohorts of *T. molitor* larvae were injected with filter-sterilized injection buffer (0.1 mM sodium phosphate, 2.5 mM potassium chloride, pH 7.2) or *dsPGRP-LE* dissolved in injection buffer or double stranded enhanced green fluorescent protein (*dsEGFP*) dissolved in injection buffer. The total amount of *dsPGRP-LE* or *dsEGFP* injected was 1 μg per larva. Injections were done using a disposable needle mounted into a micro-applicator (Picospiritzer III micro dispense system, Parker Hannifin, Hollis, NH, USA) to which the pressure was carefully adjusted so that the contents could be safely released into the hemocoel of the larvae. Larvae were injected at the 2nd or 3rd visible sternite from the posterior end of the larvae chilled on ice and handled in a dorsal-ventral position. Animals surviving the injection process were reared on diet under standard rearing conditions until the extraction of total RNA.

Larvae were evaluated for *TmPGRP-LE* gene silencing five days post injection of dsRNA. Total RNA isolation and subsequent cDNA synthesis was conducted as described under section 3.4 above. The synthesized cDNA was used as a template for semi-quantitative PCR amplification of the partial gene product using the pre-designed primers (Table 1). The PCR cycles were performed as follows: initial denaturation at 94 °C for 3 min followed by 28 cycles of denaturation at 94 °C for 30 s, annealing at 55 °C for 30 s, extension at 72 °C for 60 s and a final extension at 72 °C for 10 min in a PTC-200 thermal cycler (MJ Research, GMI, Ramsey, MN, USA). Ribosomal protein-L27A (*TmRPL27a*) gene was used as a positive control and amplified using *RPL27a* gene primers (Table 1).

Quantitative real-time PCR was performed on Exicycler™ 96 real-time quantitative thermal block (Bioneer, Korea) as previously described with the gene expression experiments. Data was analyzed by Student’s *t*-test and differences of *p* < 0.05 were considered significant.

### 3.10. Bacterial Injections and Bioassays

The bacterial cultures were grown aerobically in LB broth (for *E. coli*) and BHI broth (for *L. monocytogenes*) in an orbital shaker at 200 revolutions per minute (rpm) and 37 °C. The overnight grown cultures were washed thrice, re-suspended in sterile distilled water and serially diluted in 0.9% saline to achieve desired concentrations as determined by measurements at OD_600_. The OD_600_ values were confirmed by aseptically spread-plating the serially diluted samples on LB and BHI agar plates for *E. coli* and *L. monocytogenes*, respectively. The plates were incubated at 37 °C for 16 h prior to colony counting.

For survival studies, larvae were injected with either *dsEGFP* or *dsPGRP-LE* as described above. Five days post-injection of double strand RNA, 2 μL (~1000 CFU) of the diluted cultures was injected per larva at the 2nd or 3rd visible sternite from the posterior end of the larvae chilled on ice and handled in a dorsal-ventral position. Injections were done using a disposable needle mounted into a micro-applicator (Picospiritzer III micro dispense system, Parker Hannifin, Hollis, NH, USA) as detailed under section 3.9 above. The injected larvae were incubated at 26 °C and the number of dead larvae was recorded on a daily basis for six days post injection. Rates of survival were compared between the *PGRP-LE* silenced and (control) group. *E. coli*, which like *L. monocytogenes* is a DAP-type PGN-containing bacterium but (unlike *L. monocytogenes*) cannot survive in phagocytic cells, was included in the survival study to enable us to clearly establish the intracellular roles of PGRP-LE on the beetle immunity.

### 3.11. Statistical Analysis

Analysis of the qPCR data was done according to the 2^−ΔΔ^*^Ct^* method, where *ΔΔCt* is equal to *ΔCt* (treated sample) − *ΔCt* (control) [57]. Data from insect survival study was analyzed by the Wilcoxon-Mann Whitney test (Statistical Analysis Software, Cary, NC, USA), the cumulative survival rates (%) a *p*-value < 0.05 was considered significant.

## 4. Conclusions

We have partially characterized a PGRP-LE homologue from a coleopteran beetle *T. molitor* and attempted to evaluate its role in the survival of *T. molitor* against *L. monocytogenes* by RNAi. Although *TmPGRP-LE* RNAi did not result in significant alteration of AMP expression with or without bacterial challenge, it still caused a significant reduction in the ability of the beetle to survive *L. monocytogenes* infections. The mechanism and/or signaling cascade, by which PGRP-LE helps to enhance the survivability of the host in response to *L. monocytogenes* infection, would be central to future investigations.

## Supplementary Information



## Figures and Tables

**Table 1:** Primers sequences used in the current study.

Primer name	Sequence (5′-3′)
Oligo (dT) adaptor	GGCCACGCGTCGACTAGTACT17
M13-F (forward)	GTAAAACGACGGCCAG
M13-R (reverse)	CAGGAAACAGCTATGAC
*RpL27a* (forward)	TCATCCTGAAGGCAAAGCTCCAGT
*RpL27a* (reverse)	AGGTTGGTTAGGCAGGCACCTTTA
*TmPGRP-LE* Real-time PCR (Forward)	CTTCGCTTGCGGAATGGCAGATTA
*TmPGRP-LE* Real-time PCR (Reverse)	AACACACGCTCAAATCCTTTCCCG
*TmPGRP-LE* dsRNA (forward)	TAATACGACTCACTATAGGGAGAGGCAACGTAAATAAGGACGG
*TmPGRP-LE* dsRNA (Reverse)	TAATACGACTCACTATAGGGAGAGTAGGCGATATCGTTCCACTTC
