# Supplementary material for: Cloning, Characterization and Effect of TmPGRP-LE Gene Silencing on Survival of Tenebrio Molitor against Listeria monocytogenes Infection
Source: Int J Mol Sci. 2013 Nov 14;14(11):22462–82. doi: 10.3390/ijms141122462 (PMC3856074; doi:10.3390/ijms141122462)
Supplement: Supplementary file 1 [file ijms-14-22462-s001.pdf]

Supplementary Information

Figure S1. Predicted structural identity and features of TmPGRP-LE (**A**) secondary structure prediction for full-length TmPGRP-LE. The prediction was performed based on position-specific scoring matrices using the PSIPRED method. The sequences marked as "H", "E" and "C" correspond to helix, extended strand and random coil state, respectively. The region inside the box is representing the amidase/PGRP domain; (**B**) Predicted tertiary structure of amidase/PGRP domain of TmPGRP-LE based on homology modeling. The model was based on the reference structure of PGRP-LE in complex with tracheal cytotoxin (monomeric DAP-type PGN) [PDB id-2cb3]. The predicted structure was visualized using PyMol version 1.5. The α-helices and β-sheets are represented in red and yellow colors, respectively; and (**C**) The local model quality estimation by ANOLEA (atomic non-local environment assessment) value.

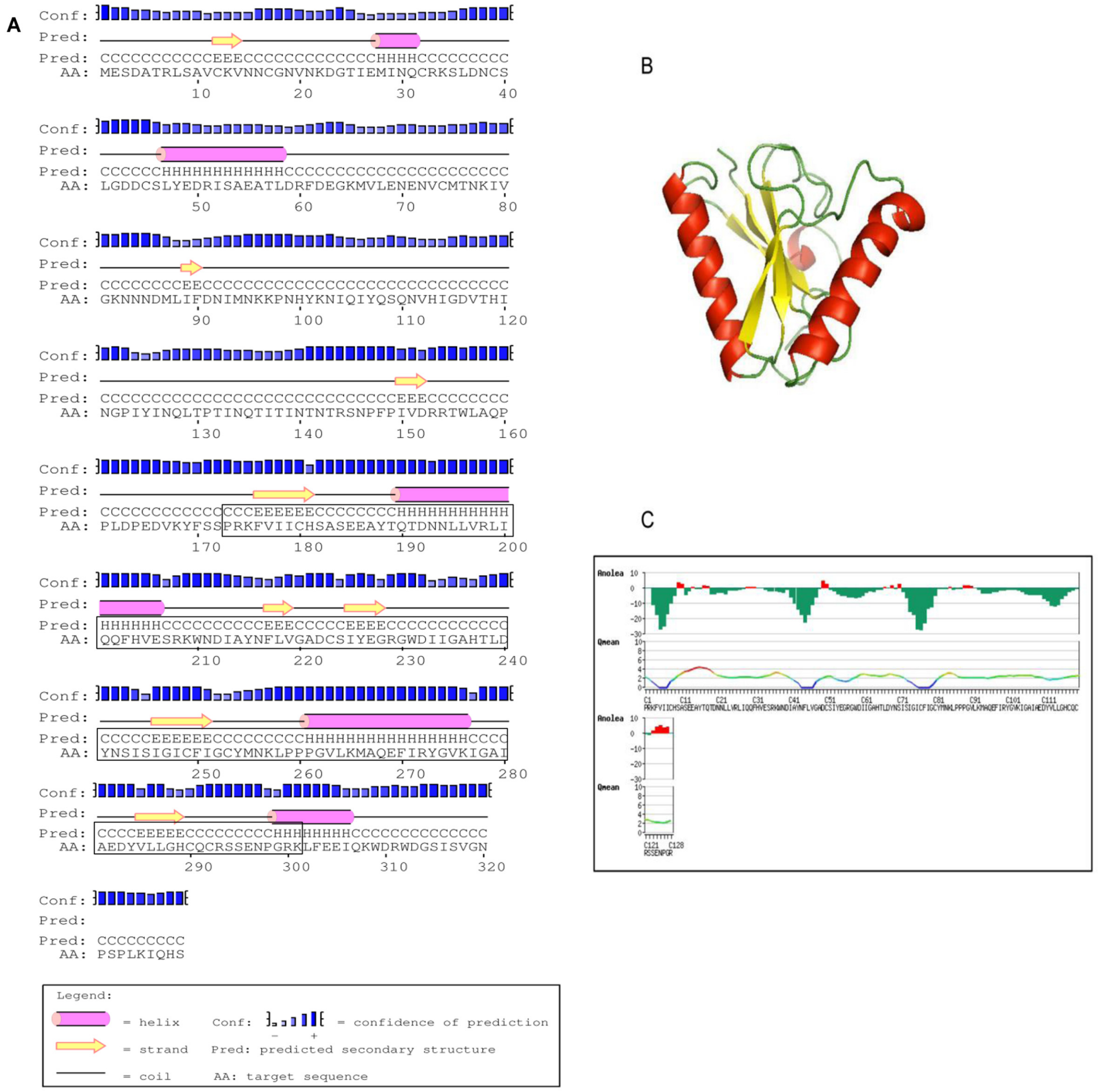

© 2013 by the authors; licensee MDPI, Basel, Switzerland. This article is an open access article distributed under the terms and conditions of the Creative Commons Attribution license (http://creativecommons.org/licenses/by/3.0/).
